# The Role of Sleep Quality, Trait Anxiety and Hypothalamic-Pituitary-Adrenal Axis Measures in Cognitive Abilities of Healthy Individuals

**DOI:** 10.3390/ijerph17207600

**Published:** 2020-10-19

**Authors:** Javier Labad, Neus Salvat-Pujol, Antonio Armario, Ángel Cabezas, Aida de Arriba-Arnau, Roser Nadal, Lourdes Martorell, Mikel Urretavizcaya, José Antonio Monreal, José Manuel Crespo, Elisabet Vilella, Diego José Palao, José Manuel Menchón, Virginia Soria

**Affiliations:** 1 Consorci Sanitari del Maresme, 08340 Mataró, Spain; jlabad@tauli.cat; 2 Institut d’Investigació i Innovació Parc Taulí (I3PT), 08208 Sabadell, Spain; jmonreal@tauli.cat (J.A.M.); dpalao@tauli.cat (D.J.P.); 3 Centro de Investigación Biomédica en Red de Salud Mental (CIBERSAM), Carlos III Health Institute, 28029 Madrid, Spain; antonio.armario@uab.cat (A.A.); roser.nadal@uab.cat (R.N.); martorelll@peremata.com (L.M.); murretavizcaya@bellvitgehospital.cat (M.U.); jmcrespo@bellvitgehospital.cat (J.M.C.); vilellae@peremata.com (E.V.); jmenchon@bellvitgehospital.cat (J.M.M.); 4 Department of Mental Health, Parc Taulí Hospital Universitari, Universitat Autònoma de Barcelona, 08208 Sabadell, Spain; nsalvat@bellvitgehospital.cat; 5 Department of Psychiatry, Bellvitge University Hospital, 08907 L’Hospitalet de Llobregat, Spain; adearriba@bellvitgehospital.cat; 6 Neurosciences Group—Psychiatry and Mental Health, Biomedical Research Institute (IDIBELL), 08908 L’Hospitalet de Llobregat, Spain; 7 Institut de Neurociències, Universitat Autònoma de Barcelona, 08193 Bellaterra, Spain; 8 Hospital Universitari Institut Pere Mata, Institut d’Investigació Sanitària Pere Virgili (IISPV), Universitat Rovira i Virgili, 43206 Reus, Spain; cabezasa@peremata.com; 9 Department of Clinical Sciences, School of Medicine, Universitat de Barcelona, 08907 L’Hospitalet de Llobregat, Spain

**Keywords:** sleep quality, memory, cortisol, trait anxiety

## Abstract

Sleep plays a crucial role in cognitive processes. Sleep and wake memory consolidation seem to be regulated by glucocorticoids, pointing out the potential role of the hypothalamic-pituitary-adrenal (HPA) axis in the relationship between sleep quality and cognitive abilities. Trait anxiety is another factor that is likely to moderate the relationship between sleep and cognition, because poorer sleep quality and subtle HPA axis abnormalities have been reported in people with high trait anxiety. The current study aimed to explore whether HPA axis activity or trait anxiety moderate the relationship between sleep quality and cognitive abilities in healthy individuals. We studied 203 healthy individuals. We measured verbal and visual memory, working memory, processing speed, attention and executive function. Sleep quality was assessed with the Pittsburgh Sleep Quality Index. Trait anxiety was assessed with the State-Trait Anxiety Inventory. HPA axis measures included the cortisol awakening response (CAR), diurnal cortisol slope and cortisol levels during the day. Multiple linear regression analyses explored the relationship between sleep quality and cognition and tested potential moderating effects by HPA axis measures and trait anxiety. Poor sleep quality was associated with poorer performance in memory, processing speed and executive function tasks. In people with poorer sleep quality, a blunted CAR was associated with poorer verbal and visual memory and executive functions, and higher cortisol levels during the day were associated with poorer processing speed. Trait anxiety was a moderator of visual memory and executive functioning. These results suggest that subtle abnormalities in the HPA axis and higher trait anxiety contribute to the relationship between lower sleep quality and poorer cognitive functioning in healthy individuals.

## 1. Introduction

Both poorer sleep quality and longer duration of sleep have been associated with impaired cognitive performance in middle-age and older adults [1,2,3]. Prospective studies have demonstrated that sleep quality disturbances are risk factors of cognitive decline [4,5,6]. Individuals with poor sleep quality are more likely to experience depressive symptoms and state and trait anxiety, as well as a negative cognitive bias (enhanced recognition of negative images) and decreased sustained attention to non-emotional stimuli [7]. The relationship between sleep problems and anxiety or depressive symptoms is thought to be bidirectional [8]. Trait anxiety, which is closely related to neuroticism and refers to the stable tendency to experience negative emotions, is a risk factor for major depression [9] and impairs the processing efficiency of manipulating working memory contents [10]. The neurobiological mechanisms linking impaired cognition and sleep quality are complex. Whether sleep, particularly rapid eye movement (REM) sleep, plays a role in neuroplasticity and memory processes is a matter of debate [11]. Some authors have suggested that during REM, new associative links are formed between memory traces already stored in the neocortex by means of enhancing the cortical plasticity involved in procedural and emotional memory [12,13]. However, other authors argue that REM sleep serves no role in the processing or consolidation of memory [14]. Sleep deprivation has also been associated with impairments in memory recall depending on the emotional content of memories, as positive and neutral memories are more susceptible to disruption by sleep loss than memories for negatively valanced material [15]. The deleterious effects of sleep deprivation on cognition appear to emerge from the interaction between the task environment and specific components of cognitive functioning [16].

There is evidence that specific neural networks that are important for memory consolidation (e.g., hippocampal and parahippocampal regions concerning visuospatial learning) are reactivated during slow-wave sleep [17]. The amygdala–hippocampus–medial prefrontal cortex network involved in emotional processing, fear, memory, and valence consolidation shows the strongest activity during REM sleep, whereas the hippocampus–medial prefrontal cortex network is more active during non-REM sleep [18]. Some authors have suggested that sleep might impact the qualitative reorganization of memory [19]: schema formation (extraction of rules that can then be generalized to novel situations) and integration (integration of recent and remote memories, relational memory and the emergence of false memories) might primarily benefit from slow-wave sleep, whereas the disintegration of a schema (disbanding existing schemas to allow “outside the box thinking” and creativity) might be facilitated by REM sleep.

Changes in synaptic strength, the primary mechanism mediating learning and memory, are thought to be influenced by the wake/sleep cycle, and a core function of sleep is to renormalize overall synaptic strength increased by waking [20]. Animal studies have demonstrated that sleep deprivation is associated with a decrease of dendritic spine numbers in hippocampal area CA1 and that recovery sleep normalizes these structural alterations [21].

Another evidence of the relevant role of sleep on cognitive processes is that sleep disorders can impair cognitive functioning. Working memory deficits have been reported in people suffering from restless leg syndrome [22] or obstructive sleep apnea [23]. However, other studies exploring different memory components in people with obstructive sleep apnea suggest that cognitive impairments affect episodic, procedural and working memory, but that these impairments are not uniform [24]. These findings underscore the need for extensive cognitive testing when exploring associations between sleep and cognition.

A potential biological mechanism explaining the role of sleep quality on cognitive impairments could be the hypothalamic-pituitary-adrenal (HPA) axis. The HPA axis is a physiological system whose activation—with the consequent release of adrenocorticotropic hormone (ACTH) from the pituitary gland and cortisol from the adrenal gland—is triggered by stress. In humans, a marked circadian rhythm in circulating levels of cortisol is observed, with higher levels during the morning and lower levels during the evening [25]. The cortisol awakening response (CAR) is a distinct facet of the circadian cortisol rhythm, which shows a physiological increase of cortisol within the first hour after awakening [26]. Cognitive impairment has been related to HPA axis dysregulation [27], including higher baseline cortisol levels [28], a blunted cortisol awakening response (CAR) and a more flattened diurnal cortisol slope [29]. Previous studies have suggested that people with poor quality sleep show increased stress reactivity of the HPA axis to physical and psychosocial stressors [30]. A recent study [31] including postmenopausal women reported different associations between sleep quality indices measured with the Pittsburgh Sleep Quality Index (PSQI) and HPA axis measures. Longer sleep latency was associated with a reduced CAR, whereas poorer subjective sleep quality, shorter sleep duration and reduced sleep efficiency were associated with a sharper cortisol decline later in the day. Other studies [32] conducted in early adolescents that measured sleep duration with actigraphy also reported associations between sleep duration and the magnitude of the CAR, with a sex-specific association, suggesting poorer sleep quality in boys was associated with lower CAR.

There is a bidirectional link between sleep and HPA axis activity. Sleep onset has an inhibitory effect on the HPA axis, which has been mostly attributed to slow-wave sleep [33]. On the other hand, acute administration of exogenous cortisol-releasing hormone (CRH) to humans is associated with sleep changes, including increased intermittent wakefulness during the total night and reduction in sleep efficiency [34]. Moreover, acute glucocorticoid administration promotes sleep intensity and suppresses REM sleep in humans [35]. Although HPA axis activity may moderate the relationship between sleep quality and cognitive performance, no previous studies have explored this issue. This issue is an important topic to be studied because sleep and wake memory consolidation seem to be regulated by glucocorticoids, with low levels of cortisol possibly facilitating the dialogue between the hippocampus and the neocortex [36].

Trait anxiety, which is closely related to neuroticism, refers to the stable tendency to experience negative emotions. This personality trait has been associated with poorer sleep quality [37] and with HPA axis abnormalities such as higher morning cortisol levels [38,39], an enhanced CAR [40], or a more flattened diurnal cortisol slope [40,41,42]. For these reasons, it is also important to explore whether trait anxiety moderates the relationship between sleep quality and cognitive abilities.

Thus, the main aim of our study was to explore whether HPA axis activity or trait anxiety moderate the relationship between sleep quality and cognitive abilities in healthy individuals. Our hypothesis was that HPA axis measures (a blunted CAR, a more flattened cortisol diurnal slope and increased cortisol levels during the day) would be associated with decreased cognitive performance, mainly in memory domains, in those individuals with poorer sleep quality. 

## 2. Materials and Methods

### 2.1. Sample

The sample consisted of 203 healthy individuals who were recruited from the general community through advertisements. All participants had no past or current history of psychiatric disorders (assessed in a semi-structured interview by an experienced psychiatrist) and a score below 7 on the 28-item Spanish adaptation of the Goldberg General Health Questionnaire (GHQ-28) [43]. The exclusion criteria were as follows: age less than 18 years, a diagnosis of psychiatric disorders including substance abuse or dependence (except nicotine), intellectual disability, neurological disorders, severe medical conditions, pregnancy or puerperium, receiving current psychopharmacological treatment (including benzodiazepines), and therapy with corticoids or contraceptive pill use in the previous three months. All participants were recruited in two provinces from Catalonia, Spain (Barcelona and Tarragona) and participated as healthy control groups in other studies exploring the role of the HPA axis on cognition in patients with major depression [44], obsessive-compulsive disorder [45] or psychotic disorders [46]. All healthy participants were recruited by consecutive sampling from the community by advertisements. A previous screening contact by email or phone call was conducted by a clinical researcher who ensured that participants did not meet any exclusion criteria before scheduling the first appointment at the hospital. The research protocol was approved by the local ethics committees of the two institutions where recruitment occurred (Bellvitge University Hospital [Ref: PR81/10] and Hospital Universitari Institut Pere Mata [Ref: 10-06-17/6proj1]). All participants provided written informed consent after having received a full explanation of the study. All procedures contributing to this work complied with the Helsinki Declaration of 1975 (revised in 2013).

### 2.2. Clinical Assessment

Sociodemographic variables and smoking habits were assessed using a semi-structured interview administered by an experienced clinician. Weight and height were measured in all participants to calculate body mass index using the formula weight (kg)/height (m^2^).

The Spanish version of the State-Trait Anxiety Inventory (STAI) [47,48] was used for assessing state and trait anxiety symptoms. The Spanish version of the STAI includes two subscales (state anxiety and trait anxiety) of 20 items that were adapted and validated to Spanish from the form X of the STAI. Each item is scored from 0–3, with a range of scores for each subscale from 0–60. The reliability of the scale in our sample was very good for both STAI state anxiety (Cronbach’s alpha = 0.87) and STAI trait anxiety (Cronbach’s alpha = 0.87).

Sleep quality was evaluated with the PSQI [49], a self-report questionnaire that assesses sleep quality over the previous month. The PSQI includes 19 self-rated questions and five questions rated by the bed partner or roommate (if available). Only the self-rated questions are included in the scoring. They measure seven different components of sleep: subjective sleep quality, sleep latency, sleep duration, habitual sleep efficiency, sleep disturbances, use of sleep medication and daytime dysfunction. The global PSQI score is calculated by adding the seven component scores, providing an overall score ranging from 0–21, where lower scores denote a healthier sleep quality. A cut-off score of 5 has been used for defining people with poor sleep quality (PSQI ≥ 5) [50]. Of all 203 participants, 22 had missing data on at least one item that did not allow us to calculate the global PSQI score. The reliability of the scale in our sample was considered adequate (Cronbach’s alpha = 0.69). All participants completed the PSQI on the same date as the clinical assessment at the hospital facilities. As most of them came alone to the visit, information on questions rated by the bed partner or roommate (item 10 subitems) were not available. 

### 2.3. Neuropsychological Assessment

The following neuropsychological tests were administered to all participants by a neuropsychologist to assess different cognitive domains (see Box S1 from Supplementary Material for additional information on each test): (1) verbal learning and memory: Hopkins Verbal Learning Test Revised (HVLT-R); (2) visual learning and memory: Brief Visuospatial Memory Test Revised (BVMT-R) and the Rey Complex Figure Test (RCFT), which includes copy, immediate recall and delayed recall sub-scores; (3) working memory: Corsi Block-Tapping Test (CBTT) for non-verbal working memory, and Letter-Number Span (LNS) to assess verbal working memory; (4) processing speed: Trail Making Test Part A (TMT-A), Brief Assessment of Cognition in Schizophrenia—Symbol Coding (BACS-SC), and category fluency (animal naming); (5) sustained attention/vigilance: Continuous Performance Test–Identical Pairs (CPT-IP); (6) selective attention/inhibition: Stroop test (direct sub-scores for words, W; colors, C; words-colors, WC; interference); (7) reasoning and problem solving: Neuropsychological Assessment Battery^®^ Mazes (NAB-Mazes); and (8) executive control: Trail Making Test Part B (TMT-B). For all but two of the cognitive tests, higher scores reflect better cognitive performance. The exceptions are the TMT-A and TMT-B, where the outcome measure is the number of seconds needed to perform the task; thus, higher scores reflect poorer cognitive performance.

Neuropsychological assessment was conducted in the morning between 9 a.m. and 12 p.m.

### 2.4. HPA Axis Measures

Saliva was collected from all participants using Salivette^®^ tubes (Sarstedt AG & Co., Nümbrecht, Germany) for cortisol determination. Five home-collected saliva samples were obtained at the following times on the same day: awakening (T1), 30′ post awakening (T2), 60′ post awakening (T3), 10 a.m. (T4), and 11 p.m. (T5). Eating, drinking, smoking or brushing teeth in the previous 15 min was not allowed. For those home-collected samples, participants were instructed to collect all samples during a regular day, avoiding stressful situations and intense physical activity. Of all premenopausal women (*N* = 69), information on the date of the last menstrual period was available for 55% of them. We calculated the difference (in days) between the dates from the last menstrual period and the HPA axis assessment to define follicular and luteal phases as described elsewhere [45]. Exploratory analyses comparing cortisol concentrations by menstrual cycle status in the subsample of women with available data were performed.

Saliva samples were processed at the Biobank of the Institut de Investigació Sanitària Pere Virgili (IISPV). After centrifugation of the Salivette tubes at 3000 rpm for 5 min, the saliva was aliquoted and frozen at −20 °C until salivary cortisol was tested in singleton using a high-sensitivity commercial chemiluminescence immunoassay (IBL, Hamburg, Germany). The intra-assay and inter-assay coefficients of variation were under 8%. The sensitivity of the assay was 0.08 nmol/L.

Three HPA axis measures were considered: CAR, diurnal cortisol slope and cortisol levels over the day.

#### 2.4.1. CAR

The CAR is a physiological response that consists of a rise in cortisol levels following morning awakening [51] and represents a distinct component of the cortisol circadian cycle, with characteristics unrelated to those of cortisol secretion throughout the rest of the day. The CAR was calculated using the area under the curve with respect to the increase (AUC_i_) with the trapezoid formula as suggested by Pruessner et al. [52]. Three cortisol concentrations (T1, T2, T3) were used for the calculation of the CAR with the AUC_i_. We also estimated the consistency of the cortisol rise after awakening as described previously [53]. Responder rates were defined as an increase of salivary cortisol levels of at least 2.5 nmol/l above baseline levels (awakening time).

#### 2.4.2. Diurnal Cortisol Slope

The HPA axis follows a marked circadian variation, with greater cortisol secretion in the morning than the evening in humans [54]. The diurnal cortisol slope reflects the rate of decline in cortisol levels across the day, from morning to evening. We calculated the slope using the T4 and T5 samples. The slope was calculated with and without transformed cortisol concentrations. In the tables, the raw slopes are displayed; however, when parametric tests and multivariate analyses were performed, slopes were calculated using transformed cortisol values (see Section 2.5).

#### 2.4.3. Cortisol Levels over the Day

The cortisol levels over the day (from awakening to 11 p.m.) were calculated using the area under the curve with respect to the ground (AUC_g_) with a trapezoid formula [52]. Although we transformed the cortisol values with a power transformation, we did not use transformed values in the calculation of cortisol levels throughout the day. This approach was preferred because the cited power transformation sometimes yields negative values for very low cortisol values (that would be the case for most evening cortisol levels), which could affect the interpretation of cortisol levels using the trapezoid formula.

### 2.5. Statistical Analyses

SPSS version 21.0 (IBM Corp., Armonk, NY, USA) was used for the statistical analyses. Cortisol values were transformed to approximate a normal distribution, as suggested by recent expert consensus guidelines [51]. The following power transformation was used: X’ = (X^0.26−1^)/0.26, as described by Miller and Plessow [55].

For descriptive purposes, we compared clinical variables between people with or without poor sleep quality taking into account the cut-off point of 5 in the PSQI. The chi-square test was used to compare categorical data among groups. T-test was used to compare continuous data. The statistical significance level was set at *p* < 0.05 (bilateral).

A univariate analysis of variance (ANOVA) was used for exploring gender and age differences in sleep quality, anxiety measures and HPA axis variables. Each continuous variable was used as the dependent variable. Gender and age group were considered independent factors. We also conducted a stratified exploratory analysis by gender and two specific age ranges: young adulthood (between 18–40 years) and middle adulthood (between 41–65 years). The association between psychometric scales, HPA axis measures and cognitive tasks was tested with Pearson’s correlations.

Multiple linear regression analyses were performed to explore the relationship between sleep quality (as a continuous measure) and cognition while testing the moderating effects of HPA axis measures and trait anxiety. All final equations were adjusted for covariates (age, gender, education level, BMI and smoking (cigarettes/day)) and potential interactions between HPA axis measures or trait anxiety and sleep quality. Cognitive tasks were considered the dependent variables. As two cognitive measures (TMT A and B) were skewed, we used natural log transformation to normalize these variables before conducting the multiple linear regression analyses. Hierarchical regressions were performed considering three models, and all independent variables were included in different steps with the enter procedure. The first model (sleep quality, unadjusted) included only the PSQI total score as the independent variable. The second model (sleep quality + HPA axis measures) included the PSQI total score and the three main HPA axis measures (CAR, cortisol diurnal slope and cortisol levels over the day), as well as cortisol levels at awakening because this measure needs to be adjusted in analyses to consider the CAR, as per the recent consensus [51]. We verified the multicollinearity of HPA axis measures with the variance inflation factor (VIF), and for all equations, VIF scores were below 2, suggesting that there was no multicollinearity. The third and final model included sleep quality scores, HPA axis measures, covariates and significant interactions. Interactions between HPA axis measures or trait anxiety and sleep quality were included with the forward procedure. Thus, only significant interactions entered the final equation. We did not adjust for state anxiety in the multivariate analyses because state anxiety and trait anxiety STAI scores in our sample were highly correlated (r = 0.59, *p* < 0.001). We aimed to avoid problems of collinearity, as trait anxiety was considered a covariate in all multiple linear regression analyses.

## 3. Results

### 3.1. Univariate Analyses

The sociodemographic and psychometric data of the sample by sleep quality groups are described in Table 1. There were no significant differences in sociodemographic variables, BMI, smoking habits or alcohol consumption. In relation to anxiety scores, those individuals with poorer sleep quality reported more trait anxiety but not more state anxiety. PSQI total scores did not differ between smokers and non-smokers, nor by participants from different alcohol groups. Regarding specific PSQI items related to potential causes of sleep problems (item 5), the prevalence of significant problems (defined as the presence of the condition related to each item during three or more times per week) were: (5a) “cannot get to sleep within 30 minutes” (11.2%); (5b) “wake up in the middle of the night or early morning” (19.3%); (5c) “have to get up to use the bathroom” (23.5%); (5d) “cannot breath comfortably” (1.6%), (5e) “cough or snore loudly” (8.6%), (5f) “feel too cold” (1.1%), (5g) “feel too hot” (10.8%), (5h) “have bad dreams” (1.1%), (5i) “have pain” (4.3%).

Cortisol measures between sleep quality groups were similar between people with good and poor sleep quality, with no significant differences in the CAR, the diurnal cortisol slope or cortisol levels over the day (Table 2). Smokers had a greater CAR when compared to non-smokers (mean [standard deviation]: 50.6 [61.6] vs. 25.1 [59.0], *p* = 0.041). No significant differences were found in other HPA axis measures between smokers and non-smokers. HPA axis measures did not differ between participants with different alcohol intake patterns (none, occasional, daily). There were no significant differences in cortisol levels nor any HPA axis measure between premenopausal women in the follicular or luteal phase. The responder rates for the CAR in the overall sample were 70.3%.

Participants with poorer sleep quality had lower scores in several cognitive tasks dealing with verbal learning, visual learning, processing speed, attention and executive function (Table 3). 

### 3.2. Exploratory Analysis by Gender and Age Range

We also explored the role of gender and age range (young adulthood versus middle adulthood) in sleep quality, anxiety and cortisol measures (Table S1). An older age was associated with poorer sleep quality, lower cortisol levels at 60′ post awakening, a more blunted CAR and lower cortisol levels over the day (Table S1; Figure S1). Sleep quality was not influenced by gender. However, women showed higher cortisol levels at awakening. A gender-by-age interaction was found in trait and state anxiety parameters, indicating that middle-aged women reported more state and trait anxiety than middle-aged men, but this gender-specific pattern was not observed in young individuals.

An exploratory heat map of correlation analyses exploring the relationship between sleep quality, anxiety, HPA axis measures and cognition in each subgroup by age and gender are described in Figures S2 and S3. Heat maps suggest that trait anxiety is more clearly associated with cognitive outcomes than state anxiety or even sleep quality. When comparing age and gender subgroups, stronger associations were found in the subgroup of middle-aged men. As shown in Figure S3, trait anxiety was associated with poorer cognitive functioning in most tasks, mainly visual memory, processing speed and executive function.

### 3.3. Multiple Linear Regression Analyses

To explore the association between sleep quality, HPA axis measures and trait anxiety with cognitive tasks while adjusting for potential confounders, independent multiple linear regression analyses were performed (Table 4; full multiple linear regression analyses are shown in Tables S2–S4).

Poor sleep quality was associated with most memory tasks (Table S2) after adjusting for HPA axis measures. However, when adjusting for clinical covariates and potential interactions, significant interactions between PSQI total scores and the CAR were found in three cognitive tasks dealing with verbal memory (HVLT-R), visual memory (BVMT-R) and working memory (LNS). These interactions suggest that there is a different pattern in the relationship between the CAR and cognitive tasks depending on reported sleep quality. In those participants with poorer sleep quality, an increased CAR was associated with better cognitive functioning. We also found that the relationship between sleep quality and cognitive performance in the BVMT-R task was moderated by trait anxiety (Table S2).

Of all processing speed tasks, sleep quality interacted with two HPA axis measures in the TMT-A (Table S3). As the TMT-A is measured in seconds, positive β coefficients reflect poorer performance on this task. A positive interaction between PSQI and cortisol levels over the day was found, suggesting that cortisol levels over the day negatively impact performance in this processing speed task, particularly in participants with poorer sleep quality. In contrast, a negative interaction between PSQI and CAR was found, which suggests that in people with poorer sleep quality, a blunted CAR was associated with poorer cognitive functioning on this task.

Finally, in those cognitive tasks dealing with attention and executive functions (Table S4), sleep quality interacted with cortisol levels over the day in two Stroop tasks (words-colors and interference scores). In participants with poorer sleep quality, higher cortisol levels during the day were associated with lower scores on both cognitive tasks, indicating poorer cognitive performance. Trait anxiety moderated the relationship between sleep quality and the TMT-B, a measure of cognitive flexibility.

## 4. Discussion

In the present study, which aimed to explore whether HPA axis activity or trait anxiety act as moderators of the relationship between sleep quality and cognitive abilities in healthy individuals, we found different roles for HPA axis measures and trait anxiety. Two HPA axis measures were moderators of the association between poor sleep quality and cognitive impairment. For instance, the CAR had a moderating effect on verbal learning, visual learning and working memory tasks, whereas cortisol levels over the day had a moderating effect on executive functioning tasks and processing speed. In general, in patients with poorer sleep quality, pathological HPA axis activity (blunted CAR, increased cortisol levels during the day) was associated with poorer cognitive functioning. Trait anxiety showed moderating effects in the relationship between sleep quality and cognition for cognitive tasks dealing with visual memory (BVMT-R) and another task that measures cognitive flexibility and has visual search demands (TMT-B). 

The results regarding the role of trait anxiety on visual cognitive tasks are in accordance with previous studies that have also reported associations between poor quality sleep and poorer cognitive performance in visual memory tasks [2] and the TMT-B [56]. Some studies have explored the relationship between trait anxiety, sleep quality and the ability to remember the extinction of conditioned fear, reporting associations between poor quality sleep and poor extinction and between poor quality sleep and trait anxiety [12]. However, this last study did not support a link between trait anxiety and poor extinction. In another study exploring the impact of trait anxiety on visual search and rapid serial visual presentation tasks [57], high-anxiety individuals allocated attention to the target less efficiently and had reduced suppression of distractors than did low-anxiety individuals. In our sample, trait anxiety was more clearly associated with impaired cognition in middle-aged men. Other studies in the literature have found gender differences in the relationship between trait anxiety and cognition, with positive correlations between neuroticism scores and verbal memory, general memory and delayed recall scores of the working memory test in healthy females [58]. Visual processing speed is also affected by sleep deprivation, which results in reduced information processing capacity [59]. Since both poor quality sleep and trait anxiety might negatively affect visual learning, common neural substrates explaining the association between sleep quality and trait anxiety may be involved in visual memory processes. In this line, the most replicated findings by structural neuroimaging studies have been negative associations between trait anxiety and grey matter volume in the prefrontal cortex, including the dorsolateral prefrontal cortex, dorsomedial prefrontal cortex and orbitofrontal cortex [60]. These brain regions, which have been implicated in sleep quality [61,62], are also critical for visual processing and learning [63].

In relation to the potential link between sleep quality and HPA axis activity on cognition, it is noteworthy that we found moderating effects of HPA axis measures for several cognitive domains that are thought to be related to hippocampus and prefrontal cortex functioning. The hippocampus, a brain area that is particularly sensitive to prolonged increases in glucocorticoid levels brought on by prolonged stress, is involved in the consolidation of declarative memory processes [27] and participates in the regulation of the HPA axis by means of negative feedback. The hippocampus is also thought to play a role in the CAR, as subjects with hippocampal damage show a blunted CAR [64]. Interestingly, the hippocampus is a key region implicated in sleep processes. Recent animal studies have compared the occurrence of sleep and sleep stages in electroencephalogram recordings of cortical activity and local field potential recordings from the prefrontal cortex and hippocampus [65]. Although slow-wave sleep congruently occurred in signals covering neocortical and hippocampal activity, REM sleep often started substantially earlier in the hippocampus than in neocortical networks. The mechanisms mediating this dissociation between hippocampal and neocortical networks are unknown. However, these findings might be relevant for the understanding of some functions, such as memory formation, that have been associated with the stage of REM sleep [11,65].

The other findings of the associations among poor sleep quality—which include cortisol levels during the day and poorer functioning in two executive functioning tasks (Stroop word-colors and interference scores) and a processing speed task (TMT-A)—point to a role of the prefrontal cortex in the relationship between sleep quality and the HPA axis. First, previous studies have related higher diurnal salivary cortisol levels with a smaller prefrontal cortex surface area in elderly men and women [66], which fits well with the potential neurotoxic effects of glucocorticoids on hippocampal neurogenesis and prefrontal cortical synaptic plasticity [67]. Second, the prefrontal cortex has been found to be involved in the Stroop task, as meta-analyses of neuroimaging studies suggest that the response to the Stroop task is highly left lateralized, most prominently in the left dorsolateral prefrontal cortex and inferior frontal regions [68]. Our findings suggest that increased cortisol levels over the day might be detrimental for some cognitive abilities related to the prefrontal cortex, particularly in those individuals who report poorer sleep quality.

In our study, all the HPA axis measures (CAR, diurnal cortisol slope and cortisol levels over the day), were not associated with poorer sleep quality. Regarding diurnal cortisol secretion patterns, previous studies have shown a flatter diurnal cortisol slope in individuals with shorter sleep [69,70] and poorer sleep quality [70,71,72]. There are mixed results in the literature regarding the CAR, with some studies reporting no relationship between sleep quality and the CAR in adolescents [73], others reporting a blunted CAR in middle-aged adults with poorer sleep quality [72] and others suggesting that poor sleep quality is associated with a blunted CAR only in police officers that are inactive or insufficiently active during their leisure time [74]. Our study is in line with the idea that CAR variation is not affected by sleep quality [75].

Although our findings suggest that HPA axis and trait anxiety moderate the relationship between HPA axis activity and memory processes, we need to underscore that we cannot infer causality due to the cross-sectional design of our study. Longitudinal studies have suggested that both sleep quality [76] and HPA axis dysregulation [77,78] are predictors of cognitive decline. However, no prospective studies have explored the interplay between sleep quality and HPA axis activity in relation to the risk of cognitive impairment over time. Future studies are needed to replicate our cross-sectional findings and to test potential mediating effects with longitudinal data. This approach would allow the exploration of whether sleep quality modifies HPA axis activity, which in turn contributes to a poorer cognitive outcome, or, on the contrary, whether a dysregulated HPA axis impairs sleep quality, leading to a poorer cognitive outcome. Prospective studies would also allow the study of potential neurobiological mechanisms involved in this process, including the determination of brain changes in regions of interest (hippocampus and prefrontal cortex). For instance, previous studies have reported greater brain atrophy and cognitive decline in healthy older adults with short sleep duration [79]. Whether these brain changes might be mediated by HPA axis activity would be interesting to explore and is particularly important because chronic stress and the associated increase in serum levels of glucocorticoids have been hypothesized to have neurotoxic effects on the brain and to negatively impact cognition [77]. Chronic stress causes remodeling of dendrites and synaptic connections in many brain regions, including not only the hippocampus but also the amygdala and medial prefrontal and orbitofrontal cortex [80].

Our study focused on the interactions between sleep quality, trait anxiety and HPA axis measures on cognitive abilities. However, other potential biological mechanisms explaining the role of poorer sleep quality on cognitive impairments include age-related changes in the hypothalamic suprachiasmatic nucleus or other important elements of the circadian timing system [81], a decline in the white matter microstructure of frontal-subcortical tracts [82], and hypoxemia and sleep fragmentation resulting from obstructive sleep apnea [83].

A limitation of our study was the assessment of sleep quality with a self-report questionnaire without the inclusion of objective measures for assessing sleep such as polysomnography or actigraphy. Nevertheless, the PSQI has been widely used in research and clinical practice, providing information on a respondent’s sleep quality [50]. Although an objective assessment of sleep might be better for exploring the quantitative aspects of sleep (i.e., sleep duration, sleep latency and number of arousals), sleep quality involves purely subjective (e.g., self-perceived) aspects such as “depth” or “restfulness” of sleep, as the assessment of sleep quality is a complex phenomenon that is difficult to measure and define objectively [50]. The PSQI was administered the same day as the cognitive testing. Therefore, sleep quality reports of each participant reflect the subjective perception of the month before the cognitive testing. We did not quantify the sleep duration or sleep quality of the day before the cognitive assessment, which might be considered a limitation. Information on the five questions rated by the partner or roommate in the PSQI were not assessed. Therefore, indirect information on potential conditions that might contribute to sleep difficulties such as restless legs syndrome or obstructive sleep apnea was not available. Moreover, only 49.5% of the sample had a roommate or bed partner. Caffeine intake of participants was not registered, and we could not adjust multivariate analyses for this variable. People with subjective complaints about sleep quality assessed with the PSQI have been suggested to have an increased risk for subthreshold levels of depressive symptoms [84]. However, the sample of our study was composed of mentally healthy individuals without a past or current history of psychiatric disorders who were interviewed by a psychiatrist and scored less than 7 points in the GHQ-28. Another limitation of our study was measuring the CAR only one day. As the original protocol also included the assessment of the dexamethasone suppression test [44,45,46], all participants took 0.25 mg of dexamethasone at 11 p.m. and therefore we dismissed the possibility of collecting further CAR sampling the next day. In the calculation of the CAR, we used the AUC with respect to the increase, as previous guidelines recommend using measures of change in cortisol levels that capture the dynamic of post-awakening cortisol changes (e.g., AUC_i_). Moreover, as the variable for cortisol levels during the day was calculated with the AUC with respect to the ground and included cortisol levels from awakening to 11 p.m., we preferred to only use the CAR as AUC_i_ for avoiding multicollinearity problems with cortisol concentrations over the day. 

Some study strengths need to be underscored. First, our study is the first to explore the interplay of distinct HPA axis measures and sleep quality on cognitive abilities in a sample of healthy individuals. Second, a detailed cognitive battery was included, which allowed the assessment of different cognitive domains including memory, executive functioning, processing speed and attention. The decision for selecting specific cognitive tests was based on the original projects that included patients with psychotic disorders and mood disorders, along with healthy individuals as a control group. Some tests that were specifically designed for patients with schizophrenia (e.g., BACS-SC) are included in the 10 cognitive tests of the MATRICS Consensus Cognitive Battery, which was initially considered the gold standard for assessing cognition in patients with schizophrenia [85]. However, over the last few years it has been widely used in patients with mood disorders [86,87]. Third, we included a sample of people without mental illnesses with a wide range of ages, including both young and middle-aged adults. Finally, we tested moderating effects of HPA axis measures and trait anxiety when exploring the relationship between sleep quality and cognition, and we also controlled for other potential confounders that might affect sleep quality such as age, gender, education level, BMI and smoking habits. 

## 5. Conclusions

Our study suggests that the role of sleep quality on cognitive abilities in healthy individuals depends on personality traits (trait anxiety) and HPA axis activity. The CAR, cortisol levels during the day and trait anxiety moderate the relationship between sleep quality and executive function and visual and working memory abilities. In people with poorer sleep quality, HPA axis alterations (blunted CAR and increased cortisol levels over the day) and high trait anxiety were associated with poorer performance in cognitive domains mediated by the hippocampus and prefrontal cortex.

## Figures and Tables

**Table 1:** Sociodemographic and clinical data of the sample by sleep quality groups.

	Total *N* = 203	Good Sleep Quality (PSQI < 5) *N* = 108	Poor Sleep Quality (PSQI ≥ 5) *N* = 73	*p* Value
Female gender	102 (56.4%)	60 (55.6%)	42 (57.5%)	0.792
Age	40.8 (17.7)	39.6 (17.6)	42.6 (17.8)	0.272
Education level (years of study)	13.3 (3.7)	13.7 (3.7)	12.7 (3.5)	0.055
Smoking	33 (18.4%)	17 (15.9%)	16 (22.2%)	0.284
Cigarettes/day (in smokers)	11.8 (10.0)	11.2 (10.4)	12.5 (9.9)	0.710
Alcohol intake				
No	99 (48.8%)	50 (46.3%)	39 (53.4%)	0.340
Occasional	76 (37.4%)	44 (40.7%)	22 (30.1%)	
Daily	28 (13.8%)	14 (13.0%)	12 (16.4%)	
BMI (kg/m^2^)	24.8 (4.8)	24.3 (4.1)	25.5 (5.6)	0.130
Working status				
Active (student or worker)	122 (67.4%)	75 (69.4%)	47 (64.4%)	0.770
Unemployed	29 (16%)	16 (14.8%)	13 (17.8%)	
Retired	30 (16.6%)	17 (15.7%)	13 (17.8%)	
Civil status				
Single	64 (35.4%)	39 (36.1%)	25 (34.2%)	0.892
Married or with stable couple	95 (52.5%)	57 (52.8%)	38 (52.1%)	
Separated or divorced	14 (7.7%)	7 (6.5%)	7 (9.6%)	
Widow	8 (4.4%)	5 (4.6%)	3 (4.1%)	
Living				
Alone	51 (28.2%)	28 (25.9%)	23 (31.5%)	0.547
With origin family	37 (20.4%)	25 (23.1%)	12 (16.4%)	
With own family or couple	77 (42.5%)	47 (43.5%)	30 (41.1%)	
With others (friends, other family)	16 (8.8%)	8 (7.4%)	8 (11.0%)	
Psychometric instruments				
PSQI total score	4.6 (2.9)	2.7 (1.1)	7.3 (2.6)	<0.001
STAI-State anxiety	11.2 (7.2)	10.8 (6.6)	11.9 (7.9)	0.303
STAI-Trait anxiety	13.9 (8.3)	12.0 (6.7)	16.9 (9.6)	<0.001

Data are mean (standard deviation) or *N* (%). Abbreviations: PSQI, Pittsburgh Sleep Quality Index; BMI, body mass index; STAI, State-Trait Anxiety Inventory.

**Table 2:** HPA axis measures by sleep quality groups.

	Total *N* = 203	Good Sleep Quality (PSQI < 5) *N* = 108	Poor Sleep Quality (PSQI ≥ 5) *N* = 73	*p* Value
Cortisol values (nmol/L)				
Cortisol at awakening	16.8 (10.5)	17.8 (11.0)	15.5 (9.6)	0.090
Cortisol 30’ post awakening	25.0 (13.5)	26.1 (13.7)	23.5 (13.2)	0.212
Cortisol 60’ post awakening	20.6 (12.5)	21.3 (13.1)	19.7 (11.4)	0.439
Cortisol at 10 a.m.	12.9 (10.3)	12.9 (11.0)	12.7 (9.3)	0.984
Cortisol at 11 p.m.	3.4 (4.1)	3.4 (5.0)	3.3 (2.4)	0.296
HPA axis measures				
CAR (AUC_i_)	31.1 (60.7)	31.0 (57.9)	31.0 (65.9)	0.996
Diurnal cortisol slope	−0.73 (0.69)	−0.72 (0.68)	−0.73 (0.69)	0.450
Cortisol levels during the day (AUC_g_)	8593.2 (5944.5)	8725.4 (6678.8)	8409.3 (4780.0)	0.737

Data are mean (standard deviation). Abbreviations: HPA, hypothalamic-pituitary-adrenal; PSQI, Pittsburgh Sleep Quality Index; CAR, cortisol awakening response, AUC_i_, area under the curve calculated with respect to the increase; AUC_g_, area under the curve calculated with respect to the ground.

**Table 3:** Cognitive assessment by sleep quality groups.

	Total *N* = 203	Good Sleep Quality (PSQI < 5) *N* = 108	Poor Sleep Quality (PSQI ≥ 5) *N* = 73	*p* Value
Verbal learning and memory				
HVLT-R	25.8 (5.0)	26.6 (5.1)	24.5 (4.7)	0.007
Visual learning and memory				
BVMT-R	25.2 (7.1)	26.3 (6.9)	23.7 (7.1)	0.016
RCFT-copy	32.6 (5.6)	32.7 (5.7)	32.4 (5.3)	0.664
RCFT-immediate recall	19.9 (7.1)	20.8 (7.1)	18.7 (6.9)	0.055
RCFT-delayed recall	20.2 (7.1)	21.1 (7.2)	18.9 (6.9)	0.041
Working memory				
CBTT (nonverbal)	15.7 (3.8)	16.1 (4.0)	15.2 (3.4)	0.138
LNS (verbal)	14.1 (3.2)	14.4 (3.0)	13.7 (3.4)	0.196
Processing speed				
TMT-A ^†^ (seconds)	36.0 (20.8)	33.9 (16.8)	29.0 (25.3)	0.104
BACS-SC	54.8 (15.1)	55.5 (15.4)	53.7 (14.7)	0.440
Category fluency	24.5 (5.9)	24.6 (5.8)	24.5 (6.1)	0.983
Stroop direct W	105.9 (16.6)	108.7 (17.0)	102.0 (15.4)	0.008
Stroop direct C	71.9 (11.8)	72.7 (11.9)	70.6 (11.6)	0.246
Attention/vigilance				
CPT-IP	2.66 (0.72)	2.8 (0.7)	2.5 (0.7)	0.022
Executive function				
TMT-B ^†^ (seconds)	69.1 (41.3)	66.1 (43.2)	73.5 (38.2)	0.085
NAB-mazes	18.2 (6.8)	18.9 (6.8)	17.0 (6.5)	0.061
Stroop direct WC	47.2 (12.2)	48.6 (11.6)	45.3 (12.9)	0.077
Stroop direct interference	4.6 (8.8)	5.2 (8.0)	3.8 (9.8)	0.291

Data are mean (standard deviation). Abbreviations: HVLT-R, Hopkins Verbal Learning Test-Revised; BVMT-R, Brief Visuospatial Memory Test-Revised; RCFT, Rey Complex Figure Test; CBTT, Corsi Block-Tapping Test; LNS, Letter Number Span; TMT-A, Trail Making Test part A; BACS-SC, Brief Assessment of Cognition in Schizophrenia-Symbol Coding; W, words; C, colors; CPT-IP, Continuous Performance Test-Identical Pairs; TMT-B, Trail Making Test part B; NAB-Mazes, Neuropsychological Assessment Battery-Mazes; WC, words-colors. ^†^ TMT-A and TMT-B raw scores are shown. *p* values calculated upon natural log-transformed variables. As both tests are measured in seconds, higher scores reflect poorer cognitive performance.

**Table 4:** Results of the multiple linear regression analyses exploring the relationship between sleep quality, hypothalamic-pituitary-adrenal axis measures, trait anxiety and cognitive functioning in healthy individuals.

	Sleep Quality (PSQI)		CAR (AUC_i_)		Diurnal Cortisol Slope		Cortisol Levels during the Day (AUC_g_)		STAI-T		Significant Interactions
	β	*p*	β	*p*	β	*p*	β	*p*	β	*p*	
Verbal learning and memory											
HVLT-R	−0.22	0.007	−0.15	0.263	−0.06	0.442	−0.11	0.188	−0.07	0.370	PSQI × CAR (β = 0.26, *p* = 0.043)
Visual learning and memory											
BVMT-R	−0.43	0.004	−0.31	0.032	−0.08	0.315	−0.04	0.645	−0.41	0.009	PSQI × CAR (β = 0.29, *p* = 0.037); PSQI × STAI-T (β = 0.49, *p* = 0.034)
RCFT-copy	0.03	0.743	0.00	0.979	−0.02	0.793	−0.02	0.866	−0.13	0.162	NS
RCFT-immediate recall	−0.08	0.298	−0.04	0.697	−0.16	0.050	−0.07	0.403	−0.17	0.039	NS
RCFT-delayed recall	−0.10	0.188	−0.02	0.823	−0.10	0.203	−0.07	0.391	−0.15	0.047	NS
Working memory											
CBTT (nonverbal)	0.03	0.699	−0.01	0.885	−0.09	0.284	−0.12	0.193	−0.02	0.812	NS
LNS (verbal)	−0.14	0.113	−0.37	0.018	−0.11	0.208	0.00	0.995	−0.12	0.157	NS
Processing speed											
TMT-A ^†^	−0.26	0.028	0.25	0.051	0.04	0.543	−0.26	0.030	0.08	0.247	PSQI × AUC_g_ all day (β = 0.51, *p* = 0.001); PSQI × CAR (β = −0.28, *p* = 0.025)
BACS-SC	0.05	0.371	−0.04	0.536	0.00	0.993	−0.03	0.592	−0.10	0.091	NS
Category fluency	0.07	0.386	−0.10	0.303	−0.15	0.091	−0.03	0.739	−0.11	0.193	NS
Stroop direct W	−0.09	0.290	0.06	0.573	0.03	0.734	0.07	0.465	−0.04	0.660	NS
Stroop direct C	0.04	0.626	−0.05	0.653	−0.04	0.677	0.02	0.866	−0.16	0.080	NS
Attention/vigilance											
CPT-IP	−0.16	0.051	0.03	0.797	0.03	0.758	0.02	0.791	0.01	0.920	NS
Executive function											
TMT-B ^†^	0.31	0.039	0.06	0.687	0.04	0.530	−0.07	0.774	0.44	0.004	PSQI × STAI-T (β = −0.55, *p* = 0.017)
NAB-mazes	−0.04	0.528	0.02	0.768	−0.09	0.211	0.04	0.641	−0.14	0.038	NS
Stroop direct WC	0.34	0.027	−0.09	0.400	−0.15	0.115	0.36	0.019	−0.09	0.312	PSQI × CAR (β = −0.52, *p* = 0.007)
Stroop direct interference	0.31	0.049	−0.13	0.243	−0.17	0.084	0.31	0.047	−0.03	0.704	PSQI × CAR (β = −0.47, *p* = 0.017)

Abbreviations: PSQI, Pittsburgh Sleep Quality Index; CAR, cortisol awakening response; AUC_i_, area under the curve calculated with respect to the increase; AUC_g_, area under the curve calculated with respect to the ground; STAI-T, State-Trait Anxiety Inventory trait anxiety subscore; HVLT-R, Hopkins Verbal Learning Test-Revised; BVMT-R, Brief Visuospatial Memory Test-Revised; RCFT, Rey Complex Figure Test; CBTT, Corsi Block-Tapping Test; LNS, Letter Number Span; TMT-A, Trail Making Test part A; BACS-SC, Brief Assessment of Cognition in Schizophrenia-Symbol Coding; CPT-IP, Continuous Performance Test-Identical Pairs; TMT-B, Trail Making Test part B; NAB-Mazes, Neuropsychological Assessment Battery-Mazes; NS, not significant. ^†^ TMT-A and TMT-B and measured in seconds. For these two cognitive tests, higher scores indicate poorer cognitive performance.

## Supplementary Materials

The following are available online at https://www.mdpi.com/1660-4601/17/20/7600/s1 Table S1. Sleep quality, anxiety and hypothalamic-pituitary-adrenal axis measures by gender and age subgroups. Table S2. Multiple linear regression analyses dealing with verbal, visual and working memory tasks. Table S3. Multiple linear regression analyses dealing with processing speed tasks. Table S4. Multiple linear regression analyses dealing with attention/vigilance and executive function tasks. Figure S1. Mean cortisol levels in healthy individuals by age and gender subgroups. Figure S2. Heat map of Pearson’s correlations between sleep quality, anxiety, HPA axis measures and cognitive tasks in young healthy individuals. Figure S3. Heat map of Pearson’s correlations between sleep quality, anxiety, HPA axis measures and cognitive tasks in middle-aged healthy individuals. Box S1. Neuropsychological tests and cognitive domains.
