# Supplementary material for: The Role of Sleep Quality, Trait Anxiety and Hypothalamic-Pituitary-Adrenal Axis Measures in Cognitive Abilities of Healthy Individuals
Source: Int J Environ Res Public Health. 2020 Oct 19;17(20):7600. doi: 10.3390/ijerph17207600 (PMC7589840; doi:10.3390/ijerph17207600)
Supplement: Supplementary file 1 [file ijerph-17-07600-s001.zip › ijerph-924800-supplementary/BoxS1_Sleep_Cognition_HPA.pdf]

Box 1. Neuropsychological tests and cognitive domains.

Verbal learning and memory

- Hopkins Verbal Learning Test Revised™ (HVLT-R): Subjects are presented with 12 words and then asked to recall as many as possible. This procedure is repeated three times. The outcome measure is the total number of words recalled, ranging from 0 to 36.

Visual learning and memory

- Brief Visuospatial Memory Test Revised™ (BVMT-R): Subjects are presented with 6 simple geometric figures for 10 seconds and then asked to recall and draw them as accurately as possible. This procedure is repeated three times. Shape (one point) and position (one point) of each of the figures are considered for the total score, which ranges from 0 to 36.
- Rey Complex Figure Test (RCFT): Subjects are asked to reproduce a complex line drawing, first by copying it (which is a measure of visuospatial constructional ability) and then from memory after a short delay (immediate recall) and then again after a 20-30 minute delay (delayed recall). Recall reproductions are measures of visual learning and memory. Each reproduction is scored taking into account the accurate position and shape of 18 design elements. The maximum score is 36.

Working memory

- Corsi Block-Tapping Test (CBTT): This test evaluates non-verbal working memory. Ten cubes arranged on a board are presented to the patient. The examiner taps pre-defined progressively longer sequences. The subject is asked to repeat the tapping sequence, forwards and then backwards. The outcome measure is the total number of correct items. Scores range from 0 to 32.
- Letter-Number Span (LNS): This is a verbal working memory task. Participants are presented with mixed letter and number sequences, increasing progressively in length. They are asked to recall items, first giving numbers in ascending order and later letters arranged alphabetically. The outcome measure is the total number of correct items and the maximum score is 24.

Processing speed

- Trail Making Test Part A (TMT-A): The task requires participants to sequentially connect 25 targets (numbers 1 to 25). The outcome measure is the number of seconds needed to perform the task, such that higher scores indicate poorer performance.
- Brief Assessment of Cognition in Schizophrenia - Symbol Coding (BACS-SC): As quickly as possible, participants write numbers 1 to 9 as matches to symbols on a response sheet for 90 seconds. The outcome measure is the total number of correct responses. This test is used not only in schizophrenia but also in affective disorders (see Cholet J, Sauvaget A, et al. Using the Brief Assessment of Cognition in Schizophrenia (BACS) to assess cognitive impairment in older patients with schizophrenia and bipolar disorder. Bipolar Disord. 2014 May; 16(3):326-36).
- Category fluency (animal naming): Participants are given 60 seconds to name as many words as possible within the animal category. The outcome measure is the number of unique words generated.

Attention/vigilance

- Continuous Performance Test - Identical Pairs (CPT-IP): The test involves a comparison between two types of stimuli: 2-digit numbers and 4-digit numbers. It requires the subject to quickly respond whenever two identical stimuli appear in a row (target) within a sequence of 300 flashed trials during a 5-minute trial. There are 60 target pairs and 60 false alarm trials in every test condition. The remaining trials, not in any way similar, are randomly organized. The primary outcome measure assesses the ability of the participant to discriminate between targets and false alarms, and provides the main index of reaction time variability. Higher scores indicate better performance.

Executive function

- Trail Making Test Part B (TMT-B): The task for participants is to sequentially connect 24 targets, alternating between numbers and letters (i.e., 1, A, 2, B, 3, etc.) using straight lines. The outcome measure is the number of seconds needed to perform the task, with higher scores indicating poorer performance.
- Neuropsychological Assessment Battery® Mazes – NAB-Mazes: These measures assess planning and organizational skills through maze-tracing tasks. There are seven mazes that become progressively more difficult. Mazes are scored based on completeness and completion time. Higher scores indicate better performance.
- Stroop: Subjects are presented with a list of color names (red, green, blue), all written in black ink, and are given 45 seconds to read as many as they can (word sub-score). A second list is then presented, containing 'Xs' written with red, green, or blue ink; the subject is now asked to give the ink color (color sub-score). A third list contains words that name colors which are different to the ink color in which the words themselves are written; the subject is again asked to give the ink color (word-color sub-score). This task requires subjects to inhibit the automatic response of reading and to name the color the word is written in. After obtaining these sub-scores, an interference score is calculated, which is the difference between real and expected interference. Positive scores indicate adequate inhibition of automatic responses, while negative scores indicate that the subject has inhibited worse than expected.
