# Supplementary material for: The Role of Sleep Quality, Trait Anxiety and Hypothalamic-Pituitary-Adrenal Axis Measures in Cognitive Abilities of Healthy Individuals
Source: Int J Environ Res Public Health. 2020 Oct 19;17(20):7600. doi: 10.3390/ijerph17207600 (PMC7589840; doi:10.3390/ijerph17207600)
Supplement: Supplementary file 1 [file ijerph-17-07600-s001.zip › ijerph-924800-supplementary/FigureS1_Sleep_Cognition_HPA.pdf]

Figure S1. Mean cortisol levels in healthy individuals by age and gender subgroups

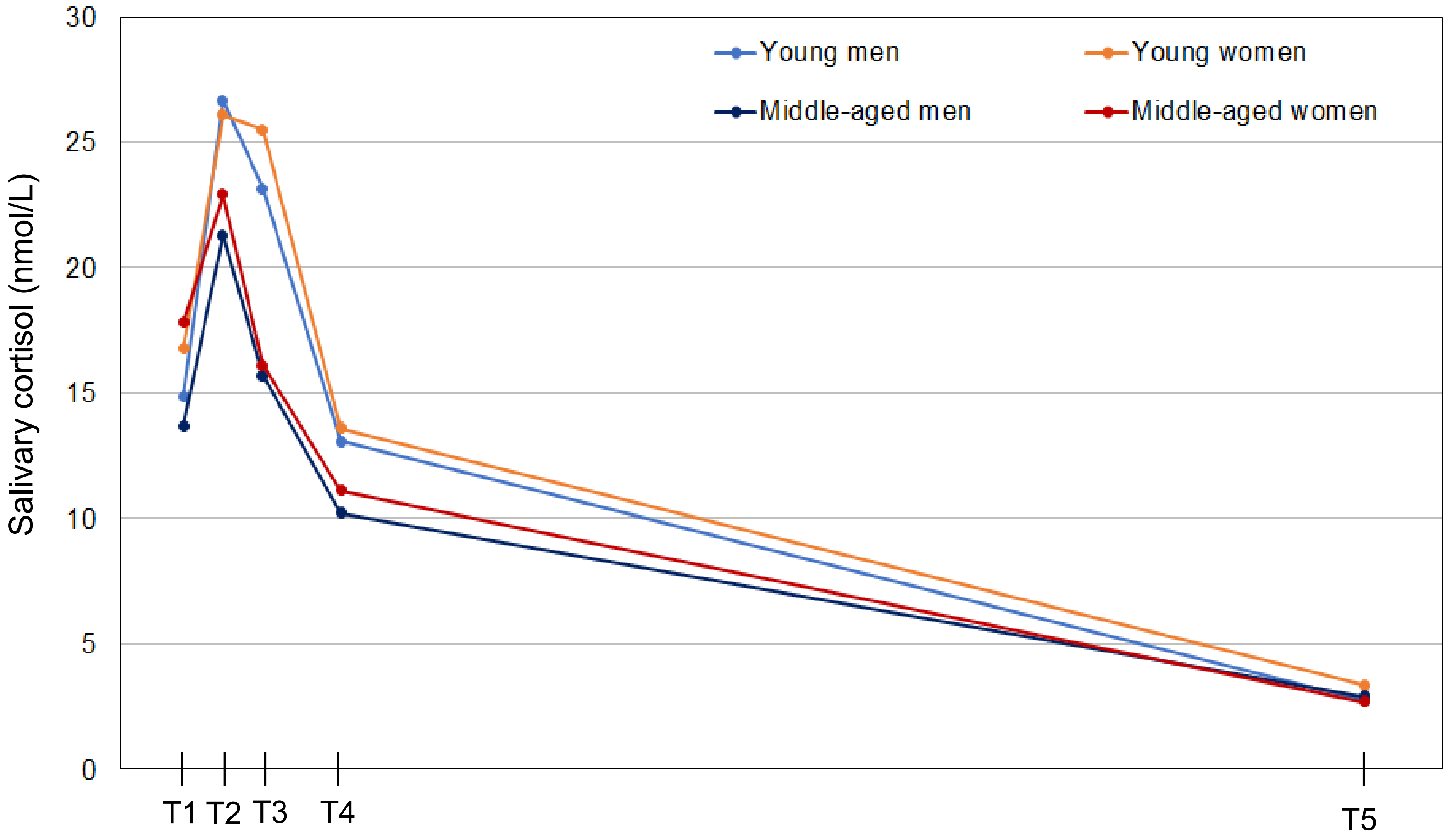

Sample points: T1: Awakening; T2: 30' Post-awakening; T3: 60'-Post Awakening; T4: 10 a.m.; T5: 11 p.m.

Age ranges: 18-40 years (young adulthood); 41-65 years (middle adulthood).
