# Supplementary material for: The Role of Sleep Quality, Trait Anxiety and Hypothalamic-Pituitary-Adrenal Axis Measures in Cognitive Abilities of Healthy Individuals
Source: Int J Environ Res Public Health. 2020 Oct 19;17(20):7600. doi: 10.3390/ijerph17207600 (PMC7589840; doi:10.3390/ijerph17207600)
Supplement: Supplementary file 1 [file ijerph-17-07600-s001.zip › ijerph-924800-supplementary/FigureS3_Sleep_Cognition_HPA.pdf]

Figure S3. Heat map of Pearson’s correlations between sleep quality, anxiety, HPA axis measures and cognitive tasks in middle-aged healthy individuals.

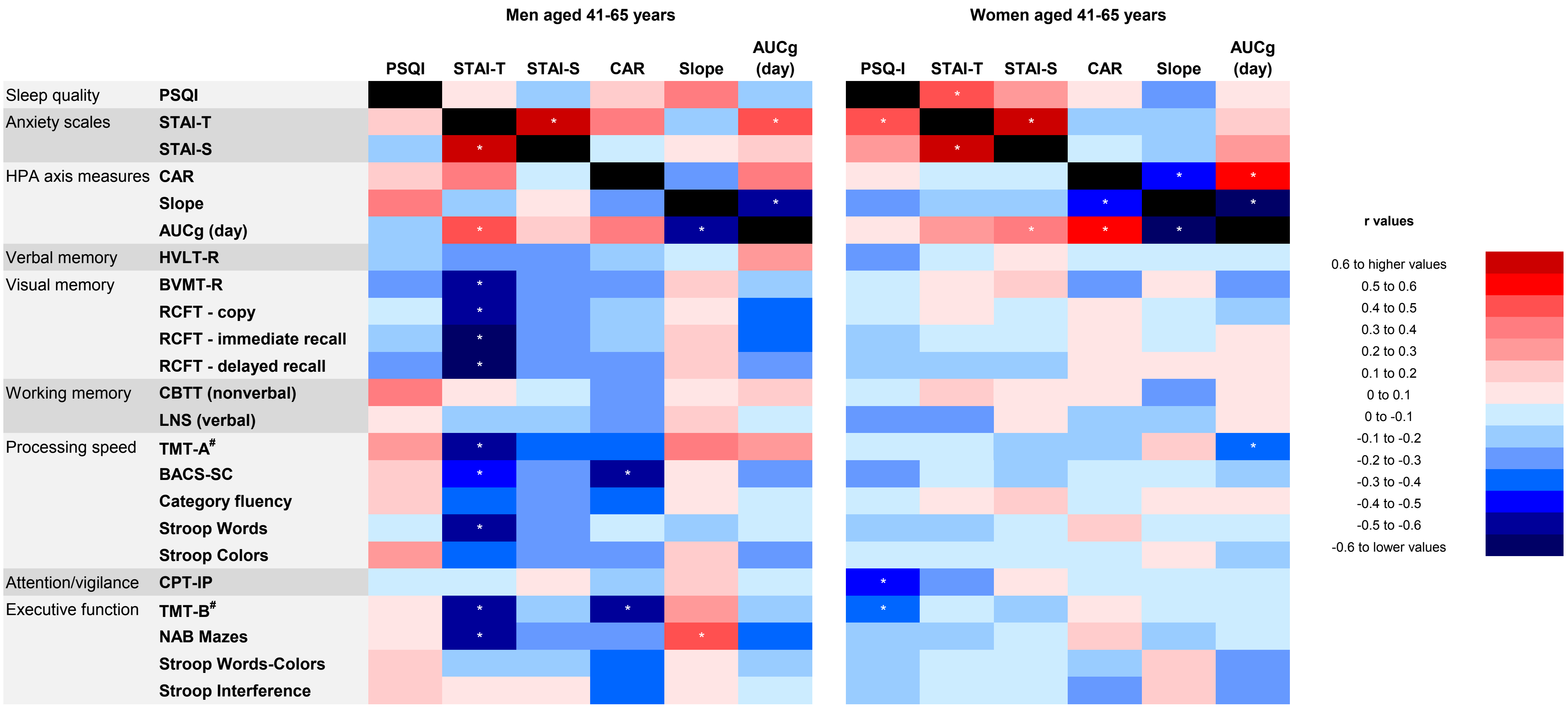

*p<0.05

#As TMT are measured in seconds, greater scores reflect poorer cognitive performance. For all other cognitive tests, higher scores reflect better cognitive performance. In the current heat map, reverse coloring was used for TMT correlations in order to make colors consistent with other tests.

Abbreviations

HPA, hypothalamic-pituitary-adrenal; PSQI, Pittsburgh Sleep Quality Index; STAI-T, State-Trait Anxiety Inventory trait subscore; STAI-S, State-Trait Anxiety Inventory state subscore; CAR, cortisol awakening response (to the increase); AUC, area under the curve; HVLT-R, Hopkins Verbal Learning Test-Revised; BVMT-R, Brief Visuospatial Memory Test-Revised; RCFT, Rey Complex Figure Test; CBTT, Corsi Block-Tapping Test; LNS, Letter Number Span; TMT-A, Trail Making Test part A; BACS-SC, Brief Assessment of Cognition in Schizophrenia-Symbol Coding; CPT-IP, Continuous Performance Test-Identical Pairs; TMT-B, Trail Making Test part B; NAB-Mazes, Neuropsychological Assessment Battery-Mazes; W, words; C, colors; WC, words-colors
