# Supplementary material for: The Role of Sleep Quality, Trait Anxiety and Hypothalamic-Pituitary-Adrenal Axis Measures in Cognitive Abilities of Healthy Individuals
Source: Int J Environ Res Public Health. 2020 Oct 19;17(20):7600. doi: 10.3390/ijerph17207600 (PMC7589840; doi:10.3390/ijerph17207600)
Supplement: Supplementary file 1 [file ijerph-17-07600-s001.zip › ijerph-924800-supplementary/TableS1_Sleep_Cognition_HPA.docx]

Table S1. Sleep quality, anxiety and hypothalamic-pituitary-adrenal axis measures by gender and age subgroups.

|  | Young men  (18-40 y)  N= 52 | Young women  (18-40 y)  N= 59 | Middle-aged men  (41-65 y)  N= 25 | Middle-aged women  (41-65 y)  N=45 | Gender effect  p | Age effect  p | Gender by age effect  p |
| --- | --- | --- | --- | --- | --- | --- | --- |
| PSQI total score | 4.0 (2.2) | 4.0 (2.8) | 4.9 (3.2) | 5.8 (3.4) | 0.249 | **0.006** | 0.282 |
| STAI-State Anxiety | 13.3 (9.1) | 9.4 (5.7) | 8.9 (5.0) | 12.8 (7.5) | 0.896 | 0.690 | **0.002** |
| STAI-Trait Anxiety | 12.5 (8.3) | 12.8 (7.3) | 11.0 (5.5) | 18.2 (9.1) | **0.009** | 0.119 | **0.017** |
| Cortisol values (nmol/L) |  |  |  |  |  |  |  |
| Cortisol at awakening | 14.9 (8.7) | 16.8 (9.1) | 13.7 (7.4) | 17.8 (8.6) | **0.033** | 0.698 | 0.390 |
| Cortisol 30' post awakening | 26.7 (14.0) | 26.1 (15.1) | 21.3 (10.0) | 22.9 (11.7) | 0.973 | 0.315 | 0.333 |
| Cortisol 60' post awakening | 23.1 (14.3) | 25.5 (13.8) | 15.7 (9.5) | 16.1 (8.4) | 0.253 | **<0.001** | 0.833 |
| Cortisol at 10 a.m. | 13.1 (10.4) | 13.6 (8.4) | 10.2 (7.4) | 11.1 (8.6) | 0.510 | 0.094 | 0.779 |
| Cortisol at 11 p.m. | 2.8 (3.2) | 3.3 (2.6) | 2.9 (2.5) | 2.7 (1.7) | 0.285 | 0.850 | 0.315 |
| HPA axis measures |  |  |  |  |  |  |  |
| CAR (AUC_i_) | 48.9 (51.0) | 39.0 (67.0) | 26.7 (61.8) | 12.3 (54.6) | 0.191 | **0.013** | 0.778 |
| Slope | -0.79 (0.67) | -0.79 (0.65) | -0.57 (0.52) | -0.65 (0.65) | 0.931 | 0.106 | 0.671 |
| Cortisol levels during the day (AUC_g_) | 8579.9 (5994.3) | 9088.4 (4625.1) | 6971.2 (4014.4) | 7397.1 (4083.7) | 0.621 | **0.044** | 0.891 |

Abbreviations: Abbreviations: PSQI, Pittsburgh Sleep Quality Index; STAI, State-Trait Anxiety Inventory; CAR, cortisol awakening response; AUC_i_, area under the curve calculated with respect to the increase; AUC_g_, area under the curve calculated with respect to the ground.
