# Supplementary material for: The Role of Sleep Quality, Trait Anxiety and Hypothalamic-Pituitary-Adrenal Axis Measures in Cognitive Abilities of Healthy Individuals
Source: Int J Environ Res Public Health. 2020 Oct 19;17(20):7600. doi: 10.3390/ijerph17207600 (PMC7589840; doi:10.3390/ijerph17207600)
Supplement: Supplementary file 1 [file ijerph-17-07600-s001.zip › ijerph-924800-supplementary/TableS2_Sleep_Cognition_HPA.docx]

|  | HVLTR | | BVMT | | RCF Copy | | RCF Immediate | | RCF Delayed | | CBTT | | LNS | |
| --- | --- | --- | --- | --- | --- | --- | --- | --- | --- | --- | --- | --- | --- | --- |
| Independent variables | β | p | β | p | β | p | β | p | β | p | β | p | β | p |
| **Model 1 (unadjusted)** |  |  |  |  |  |  |  |  |  |  |  |  |  |  |
| PSQI total score | -0.24 | 0.002 | -0.23 | 0.005 | -0.07 | 0.368 | -0.23 | 0.004 | -0.26 | 0.001 | -0.08 | 0.318 | -0.19 | 0.017 |
| **Model 2 (PSQI + HPA axis measures)** |  |  |  |  |  |  |  |  |  |  |  |  |  |  |
| PSQI total score | -0.24 | 0.002 | -0.23 | 0.005 | -0.07 | 0.396 | -0.22 | 0.005 | -0.25 | 0.002 | -0.08 | 0.337 | -0.20 | 0.016 |
| Cortisol at awakening | 0.04 | 0.723 | -0.05 | 0.648 | 0.01 | 0.957 | -0.03 | 0.746 | 0.01 | 0.947 | 0.02 | 0.846 | -0.09 | 0.385 |
| Diurnal cortisol slope | -0.12 | 0.191 | -0.12 | 0.182 | -0.06 | 0.499 | -0.19 | 0.031 | -0.14 | 0.108 | -0.13 | 0.156 | -0.08 | 0.386 |
| CAR (AUC_i_) | 0.23 | 0.024 | 0.07 | 0.475 | 0.10 | 0.364 | 0.09 | 0.379 | 0.12 | 0.225 | 0.12 | 0.271 | -0.03 | 0.766 |
| AUC_g_ all day | -0.17 | 0.076 | -0.09 | 0.349 | -0.06 | 0.569 | -0.12 | 0.217 | -0.12 | 0.211 | -0.15 | 0.132 | -0.01 | 0.924 |
| **Model 3 (PSQI + HPA axis measures + covariates and interactions)** |  |  |  |  |  |  |  |  |  |  |  |  |  |  |
| PSQI total score | -0.22 | 0.007 | -0.43 | 0.004 | 0.03 | 0.743 | -0.08 | 0.298 | -0.10 | 0.188 | 0.03 | 0.699 | -0.14 | 0.113 |
| Cortisol at awakening | 0.04 | 0.622 | -0.03 | 0.706 | 0.01 | 0.890 | -0.03 | 0.779 | 0.02 | 0.796 | 0.05 | 0.637 | -0.11 | 0.273 |
| Diurnal cortisol slope | -0.06 | 0.442 | -0.08 | 0.315 | -0.02 | 0.793 | -0.16 | 0.050 | -0.10 | 0.203 | -0.09 | 0.284 | -0.11 | 0.208 |
| CAR (AUC_i_) | -0.15 | 0.263 | -0.31 | 0.032 | 0.00 | 0.979 | -0.04 | 0.697 | -0.02 | 0.823 | -0.01 | 0.885 | -0.37 | 0.018 |
| AUC_g_ all day | -0.11 | 0.188 | -0.04 | 0.645 | -0.02 | 0.866 | -0.07 | 0.403 | -0.07 | 0.391 | -0.12 | 0.193 | 0.00 | 0.995 |
| Age | -0.46 | <0.001 | -0.38 | <0.001 | -0.31 | 0.004 | -0.34 | <0.001 | -0.42 | <0.001 | -0.31 | 0.003 | -0.07 | 0.486 |
| Education level | 0.24 | 0.001 | 0.25 | 0.002 | 0.07 | 0.451 | 0.25 | 0.002 | 0.29 | <0.001 | 0.25 | 0.003 | 0.37 | <0.001 |
| Female gender | 0.19 | 0.005 | -0.03 | 0.731 | 0.04 | 0.620 | -0.04 | 0.582 | -0.07 | 0.339 | -0.08 | 0.288 | -0.02 | 0.835 |
| STAI-Trait | -0.07 | 0.370 | -0.41 | 0.009 | -0.13 | 0.162 | -0.16 | 0.039 | -0.15 | 0.047 | -0.02 | 0.812 | -0.12 | 0.157 |
| BMI | 0.09 | 0.256 | 0.10 | 0.274 | 0.06 | 0.527 | 0.20 | 0.025 | 0.25 | 0.003 | 0.01 | 0.947 | -0.06 | 0.507 |
| Smoking (cig/day) | 0.04 | 0.600 | -0.09 | 0.300 | -0.07 | 0.375 | -0.05 | 0.512 | -0.06 | 0.372 | 0.02 | 0.802 | -0.05 | 0.484 |
| Interaction PSQI x CAR | 0.26 | 0.043 | 0.29 | 0.037 |  |  |  |  |  |  |  |  | 0.32 | 0.028 |
| Interaction PSQI x STAI-Trait |  |  | 0.49 | 0.034 |  |  |  |  |  |  |  |  |  |  |

Table S2. Multiple linear regression analyses dealing with verbal, visual and working memory tasks.

Abbreviations: PSQI, Pittsburgh Sleep Quality Index; HPA, hypothalamic-pituitary-adrenal axis; CAR, cortisol awakening response; AUC_i_, area under the curve calculated with respect to the increase; AUC_g_, area under the curve calculated with respect to the ground; STAI-Trait, State-Trait Anxiety trait subscore; BMI, body mass index; HVLT-R, Hopkins Verbal Learning Test-Revised; BVMT-R, Brief Visuospatial Memory Test-Revised; RCFT, Rey Complex Figure Test; CBTT, Corsi Block-Tapping Test; LNS, Letter Number Span; TMT-A, Trail Making Test part A; BACS-SC, Brief Assessment of Cognition in Schizophrenia-Symbol Coding; CPT-IP, Continuous Performance Test-Identical Pairs; TMT-B, Trail Making Test part B; NAB-Mazes, Neuropsychological Assessment Battery-Mazes; W, words; C, colours; WC, words-colours.
