# Supplementary material for: The Role of Sleep Quality, Trait Anxiety and Hypothalamic-Pituitary-Adrenal Axis Measures in Cognitive Abilities of Healthy Individuals
Source: Int J Environ Res Public Health. 2020 Oct 19;17(20):7600. doi: 10.3390/ijerph17207600 (PMC7589840; doi:10.3390/ijerph17207600)
Supplement: Supplementary file 1 [file ijerph-17-07600-s001.zip › ijerph-924800-supplementary/TableS3_Sleep_Cognition_HPA.docx]

| Independent variables | TMT-A | | BACS-SC | | Fluency | | Stroop Words | | Stroop Colours | |
| --- | --- | --- | --- | --- | --- | --- | --- | --- | --- | --- |
|  | β | p | β | p | β | p | β | p | β | p |
| **Model 1 (unadjusted)** |  |  |  |  |  |  |  |  |  |  |
| PSQI total score | 0.16 | 0.049 | -0.12 | 0.125 | -0.04 | 0.612 | -0.18 | 0.030 | -0.03 | 0.708 |
| **Model 2 (PSQI + HPA axis measures)** |  |  |  |  |  |  |  |  |  |  |
| PSQI total score | 0.16 | 0.045 | -0.13 | 0.095 | -0.04 | 0.593 | -0.18 | 0.030 | -0.04 | 0.609 |
| Cortisol at awakening | 0.05 | 0.611 | -0.10 | 0.340 | -0.07 | 0.528 | -0.07 | 0.512 | -0.13 | 0.239 |
| Cortisol diurnal slope | 0.09 | 0.304 | -0.08 | 0.361 | -0.13 | 0.168 | 0.02 | 0.866 | -0.02 | 0.806 |
| CAR (AUC_i_) | -0.16 | 0.115 | 0.18 | 0.085 | -0.01 | 0.898 | 0.14 | 0.186 | 0.00 | 0.972 |
| AUC_g_ all day | 0.10 | 0.305 | -0.12 | 0.245 | -0.07 | 0.479 | 0.05 | 0.594 | -0.01 | 0.958 |
| **Model 3 (PSQI + HPA axis measures + covariates and interactions)** |  |  |  |  |  |  |  |  |  |  |
| PSQI total score | -0.26 | 0.028 | 0.05 | 0.371 | 0.07 | 0.386 | -0.09 | 0.290 | 0.04 | 0.626 |
| Cortisol at awakening | -0.02 | 0.768 | -0.07 | 0.275 | -0.10 | 0.303 | -0.06 | 0.580 | -0.14 | 0.184 |
| Cortisol diurnal slope | 0.04 | 0.543 | 0.00 | 0.993 | -0.15 | 0.091 | 0.03 | 0.734 | -0.04 | 0.677 |
| CAR (AUC_i_) | 0.25 | 0.051 | -0.04 | 0.536 | -0.09 | 0.360 | 0.06 | 0.568 | -0.05 | 0.653 |
| AUC_g_ all day | -0.26 | 0.030 | -0.03 | 0.592 | -0.03 | 0.739 | 0.07 | 0.465 | 0.02 | 0.866 |
| Age | 0.72 | <0.001 | -0.60 | <0.001 | -0.05 | 0.644 | -0.17 | 0.115 | -0.04 | 0.718 |
| Education level | -0.10 | 0.121 | 0.35 | <0.001 | 0.43 | <0.001 | 0.14 | 0.117 | 0.05 | 0.570 |
| Female gender | 0.04 | 0.488 | 0.06 | 0.256 | 0.07 | 0.347 | -0.02 | 0.774 | 0.05 | 0.584 |
| STAI-Trait | 0.08 | 0.247 | -0.10 | 0.091 | -0.11 | 0.193 | -0.04 | 0.660 | -0.16 | 0.080 |
| BMI | -0.09 | 0.211 | 0.07 | 0.296 | 0.08 | 0.369 | -0.09 | 0.344 | -0.11 | 0.278 |
| Smoking (cig/day) | 0.05 | 0.458 | -0.09 | 0.121 | -0.01 | 0.882 | -0.05 | 0.544 | 0.00 | 0.987 |
| Interaction PSQI x AUC_g_ all day | 0.51 | 0.001 |  |  |  |  |  |  |  |  |
| Interaction PSQI x CAR | -0.28 | 0.025 |  |  |  |  |  |  |  |  |

Table S3. Multiple linear regression analyses dealing with processing speed tasks.

Abbreviations: PSQI, Pittsburgh Sleep Quality Index; CAR, cortisol awakening response to the increase; AUC_i_, area under the curve calculated with respect to the increase; AUC_g_, area under the curve calculated with respect to the ground; STAI-Trait, State-Trait Anxiety trait subscore; BMI, Body mass index; TMT-A, Trail Making Test part A; BACS-SC, Brief Assessment of Cognition in Schizophrenia-Symbol Coding.
