# Supplementary material for: The Role of Sleep Quality, Trait Anxiety and Hypothalamic-Pituitary-Adrenal Axis Measures in Cognitive Abilities of Healthy Individuals
Source: Int J Environ Res Public Health. 2020 Oct 19;17(20):7600. doi: 10.3390/ijerph17207600 (PMC7589840; doi:10.3390/ijerph17207600)
Supplement: Supplementary file 1 [file ijerph-17-07600-s001.zip › ijerph-924800-supplementary/TableS4_Sleep_Cognition_HPA.docx]

| Independent variables | CPT-IP | | TMT-B | | NAB Mazes | | Stroop Words-Colours | | Stroop Interference | |
| --- | --- | --- | --- | --- | --- | --- | --- | --- | --- | --- |
|  | β | p | β | p | β | p | β | p | β | p |
| **Model 1 (unadjusted)** |  |  |  |  |  |  |  |  |  |  |
| PSQI total score | -0.22 | 0.007 | 0.16 | 0.048 | -0.22 | 0.006 | -0.11 | 0.193 | -0.08 | 0.354 |
| **Model 2 (PSQI + HPA axis measures)** |  |  |  |  |  |  |  |  |  |  |
| PSQI total score | -0.24 | 0.004 | 0.16 | 0.052 | -0.22 | 0.005 | -0.11 | 0.185 | -0.07 | 0.365 |
| Cortisol at awakening | -0.10 | 0.355 | 0.06 | 0.540 | -0.14 | 0.157 | -0.11 | 0.293 | -0.07 | 0.493 |
| Cortisol diurnal slope | 0.03 | 0.749 | 0.08 | 0.390 | -0.15 | 0.076 | -0.10 | 0.280 | -0.13 | 0.174 |
| CAR (AUC_i_) | 0.08 | 0.422 | -0.11 | 0.291 | 0.19 | 0.051 | -0.05 | 0.637 | -0.11 | 0.311 |
| AUC_g_ all day | 0.01 | 0.960 | 0.01 | 0.940 | 0.00 | 0.983 | 0.02 | 0.856 | 0.01 | 0.907 |
| **Model 3 (PSQI + HPA axis measures + covariates and interactions)** |  |  |  |  |  |  |  |  |  |  |
| PSQI total score | -0.16 | 0.051 | 0.31 | 0.039 | -0.04 | 0.528 | 0.34 | 0.027 | 0.31 | 0.049 |
| Cortisol at awakening | -0.10 | 0.289 | 0.07 | 0.443 | -0.09 | 0.262 | -0.10 | 0.319 | -0.06 | 0.565 |
| Cortisol diurnal slope | 0.03 | 0.758 | 0.04 | 0.530 | -0.09 | 0.211 | -0.15 | 0.115 | -0.17 | 0.084 |
| CAR (AUC_i_) | 0.03 | 0.797 | 0.06 | 0.687 | 0.02 | 0.768 | -0.09 | 0.400 | -0.13 | 0.243 |
| AUC_g_ all day | 0.02 | 0.791 | -0.07 | 0.774 | 0.04 | 0.641 | 0.36 | 0.019 | 0.31 | 0.047 |
| Age | -0.12 | 0.237 | 0.33 | 0.001 | -0.52 | <0.001 | -0.12 | 0.244 | -0.09 | 0.392 |
| Education level | 0.39 | <0.001 | -0.29 | <0.001 | 0.14 | 0.047 | 0.18 | 0.039 | 0.18 | 0.047 |
| Female gender | -0.09 | 0.259 | 0.02 | 0.970 | -0.18 | 0.005 | 0.01 | 0.867 | 0.00 | 0.953 |
| STAI-Trait | 0.01 | 0.920 | 0.44 | 0.004 | -0.14 | 0.038 | -0.09 | 0.312 | -0.03 | 0.704 |
| BMI | 0.08 | 0.383 | 0.04 | 0.622 | 0.09 | 0.247 | -0.07 | 0.469 | -0.02 | 0.845 |
| Smoking (cig/day) | -0.16 | 0.044 | 0.02 | 0.764 | -0.08 | 0.214 | -0.06 | 0.447 | -0.07 | 0.399 |
| Interaction PSQI x STAI-Trait |  |  | -0.55 | 0.017 |  |  |  |  |  |  |
| Interaction PSQI x AUC_g_ all day |  |  |  |  |  |  | -0.52 | 0.007 | -0.47 | 0.017 |

Table S4. Multiple linear regression analyses dealing with attention/vigilance and executive function tasks.

Abbreviations: PSQI, Pittsburgh Sleep Quality Index; HPA, hypothalamic-pituitary-adrenal axis; CAR, cortisol awakening response; AUC_i_, area under the curve calculated with respect to the increase; AUC_g_, area under the curve calculated with respect to the ground; STAI-Trait, State-Trait Anxiety trait subscore; BMI, Body mass index; CPT-IP, Continuous Performance Test-Identical Pairs; TMT-B, Trail Making Test part B; NAB-Mazes, Neuropsychological Assessment Battery-Mazes.
